# Psip1/Ledgf p52 Binds Methylated Histone H3K36 and Splicing Factors and Contributes to the Regulation of Alternative Splicing

**DOI:** 10.1371/journal.pgen.1002717

**Published:** 2012-05-17

**Authors:** Madapura M. Pradeepa, Heidi G. Sutherland, Jernej Ule, Graeme R. Grimes, Wendy A. Bickmore

**Affiliations:** 1 MRC Human Genetics Unit, MRC Institute of Genetics and Molecular Medicine, University of Edinburgh, Edinburgh, United Kingdom; 2 MRC Laboratory of Molecular Biology, Cambridge, United Kingdom; The Babraham Institute, United Kingdom

## Abstract

Increasing evidence suggests that chromatin modifications have important roles in modulating constitutive or alternative splicing. Here we demonstrate that the PWWP domain of the chromatin-associated protein Psip1/Ledgf can specifically recognize tri-methylated H3K36 and that, like this histone modification, the Psip1 short (p52) isoform is enriched at active genes. We show that the p52, but not the long (p75), isoform of Psip1 co-localizes and interacts with Srsf1 and other proteins involved in mRNA processing. The level of H3K36me3 associated Srsf1 is reduced in Psip1 mutant cells and alternative splicing of specific genes is affected. Moreover, we show altered Srsf1 distribution around the alternatively spliced exons of these genes in Psip1 null cells. We propose that Psip1/p52, through its binding to both chromatin and splicing factors, might act to modulate splicing.

## Author Summary

The regulated processing of mRNAs by splicing of exons and introns has the potential to increase the information content of the genome. Various splicing factors have been identified whose binding to cis-acting sequences can influence whether an alternative exon is included or excluded (skipped) in the mature mRNA. However, increasing evidence suggests that the chromatin template also has an important role in modulating splicing. Here we identify a chromatin-associated protein Psip1/Ledgf that can bind to a histone modification enriched at active genes and that can also interact with other proteins involved in mRNA splicing. Loss of Psip1 reduces the chromatin association of specific splicing proteins and alters the pattern of alternative splicing. We propose that Psip1, through its binding to both chromatin and splicing factors, might act to modulate splicing.

## Introduction

Pre-mRNA splicing occurs co-transcriptionally [1], whilst the nascent transcript is still associated with the chromatin template. However, until recently there has been little consideration of how chromatin structure might influence the control of splicing. Initial studies indicated a link between promoters and alternative splicing [2]–[4] and this has been extended to histone modifications enriched at promoters. For example, Gcn5 mediated histone acetylation at promoters in yeast has been shown to facilitate recruitment of splicing factors [5] and mammalian GCN5-containing complexes interact with pre-mRNA splicing factors [6]. The chromatin remodeller CHD1, which recognises a histone mark (H3K4me3) enriched at active promoters, also interacts with spliceosome components and affects the rate of mRNA splicing [7].

A link between the rate of transcriptional elongation and splicing [8]–[10] has led to a consideration of how chromatin structure within the body of genes might also influence splicing. Increased levels of histone acetylation in gene bodies lead to exon skipping, likely through enhanced RNA polymerase II processivity [11]. Conversely, HP1γ, which binds to H3K9me3, favors inclusion of alternative exons, possibly by decreasing RNA polymerase II elongation rate [12].

Trimethylation of H3 at lysine 36 (H3K36me3) is enriched at exons, particularly those of highly expressed genes [13]– and its level at alternatively spliced exons is reported to correlate with their inclusion into the spliced transcript [13]. An explanation for this may come from observations that pre-mRNA splicing itself affects the deposition of this histone modification [18], [19]. A direct link between H3K36me3 and an effect on mRNA splicing comes from the observation that MRG15, a protein whose chromodomain can recognise H3K36me3, recruits polypyrimidine tract binding protein (PTB) to alternatively spliced exons [20]. It was not clear whether this is a unique interaction or whether there are other systems that connect H3K36me3 to alternative splicing.

**Figure 1:**
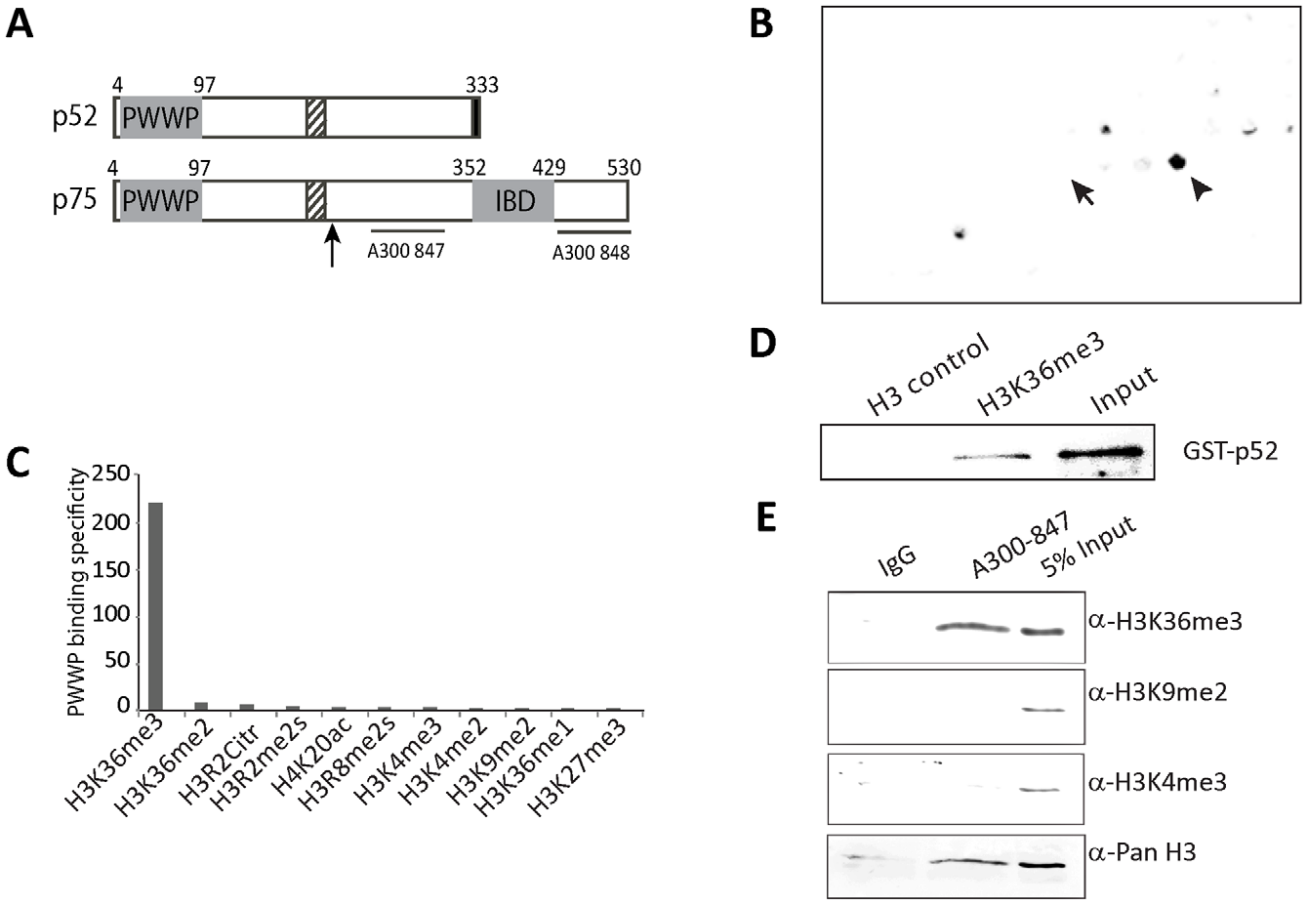
Psip1 PWWP domain binds to H3K36me3. A) Diagram of p52 and p75 Psip1 isoforms showing the position of the; PWWP domain, AT hook-like domains (hatched box), C-terminal 8 a.a. unique to p52 (black box), and the p75-specific IBD. Vertical arrow indicates the site of gene trap integration in *Psip1^gt/gt^* [22]. Horizontal lines indicate the position of epitopes recognized by antibodies A300-847 and A300-848. B) Peptide array containing 384 histone tail modification combinations incubated with GST-Psip1-PWWP and detected with αGST. Spots corresponding to unmodified H3 26–45 peptide (arrow) and H3K36me3 (arrowhead) are indicated. C) Binding specificity (calculated from the intensity of the histone peptide interaction) of Psip1-PWWP (y axis) to the top list of histone modifications arranged according to decreasing specificity (x axis). Data for all the modifications are provided in Table S1. D) Immunoblot of biotinylated H3K36me3 peptide pull-down detecting GST-p52 with αGST antibodies. Corresponding unmodified histone H3 peptide served as control and GST-p52 was loaded as input. E) Immunoblot of A300-847 IPs with antibodies detecting; unmodified H3, H3K36me3, H3K9me2 and H3K4me3. IgG served as control and 5% of NIH3T3 nuclear extract was loaded as input.

PC4 and SF2 interacting protein 1 (Psip1) has been implicated in transcriptional regulation and mRNA splicing in vitro [21], but its function in vivo is poorly understood. It has been implicated in developmental gene regulation [22] and in guiding the integration of human immunodeficiency virus (HIV) into the host genome [23]–[26]. *Psip1* encodes two protein isoforms - p52 and p75 - generated by alternative splicing within intron 9, and whose relative levels vary between tissues [21], [27]. The p75 isoform, also known as lens epithelium derived growth factor (Ledgf), has a C-terminal integrase binding domain (IBD) (Figure 1A) that binds the integrases of HIV-1 and other lentiviruses, preventing their degradation by the proteosome [28] and tethering them to host chromosomes [28]–[33]. In Psip1 mutant cells, HIV/lentivirus infection is impaired and sites of viral integration into the host genome are altered [24]–[26]. Though the normal cellular function of Psip1/p75 has not been established, the IBD binds to RAM2/JPO2 - a myc-associated transcriptional regulator [34], [35] and p75 is tethered, via Menin and in an IBD-dependant manner, to MLL H3K4 histone methyltransferase [36].

The p52 isoform of Psip1 lacks the IBD (Figure 1A) and does not interact with Menin. Instead, Psip1/p52 has been purified with PC4 transcriptional co-activator [37], and had been shown to immunoprecipitate (IP) with, and to modulate the activity of, the splicing factor SRSF1 (previously known as SF2/ASF) [38], indicating that it might have a role in mRNA processing.

Here we show that the N-terminal PWWP domain, common to both Psip1 isoforms, can specifically recognize H3K36me3 and, that like H3K36me3, Psip1/p52 is enriched at expressed genes and often at the downstream exons of those genes. We demonstrate that Psip1/p52, but not p75, also interacts with proteins known to be involved in splicing and RNA processing and co-localizes with splicing factor-enriched speckles in the nucleus. Furthermore, we show that there is altered alternative splicing in Psip1 mutant cells and that this is attributable to loss of function of the p52 isoform. We demonstrate altered association of Srsf1 with the genome in the absence of functional Psip1, including around some exons whose inclusion or exclusion into mRNA is altered in Psip1 mutant cells. We propose that Psip1/p52 provides a new example of communication between chromatin and the regulation of mRNA splicing.

## Results

### Psip1 PWWP domain can bind to H3K36me3

GFP-tagged full-length, and β-gal tagged gene-trap, versions of Psip1/p75 have been reported on mitotic chromosomes [22], [30], [39], [40]. The N-terminal PWWP (Pro-Trp-Trp-Pro) domain (Figure 1A) is required for chromatin association [41]. PWWP belongs to the Tudor (Royal) family of protein domains, which are known to bind methylated lysines, including in histones [42] and the PWWP domains of Brpf1, Dnmt3a, MSH-6, NSD1, NSD2 and N-PAC have been shown to specifically bind H3K36me3 [43]–[45].

To determine if the Psip1 PWWP domain directly interacts with modified histone tails, we used histone tail peptide arrays containing in total 59 different modifications of H3, H4, H2A, and H2B tails in 384 different combinations. In two independent experiments, we observed that GST-tagged Psip1 PWWP domain bound H3K36me3 with high specificity - signal from H3K36me2, H3K36me and corresponding unmodified peptide spots were not above background (Figure 1B and 1C, Table S1). Direct binding of p52 with H3K36me3 was confirmed by peptide pulldown (Figure 1D). Immunoblotting with antibodies recognizing different H3 methylation states confirmed a specific enrichment of H3K36me3 in Psip1 IPs from nuclear extracts (Figure 1E).

### Psip1/p52 is enriched at expressed genes

We assessed the genomic distribution of Psip1 in mouse embryonic fibroblasts (MEFs) by chromatin immunoprecipitation (ChIP) using αPsip1 antibody A300-847 (see below) and hybridization to a custom tiling array. The hybridization pattern was compared to that from H3K36me3 and H3K4me3 ChIPs.

**Figure 2:**
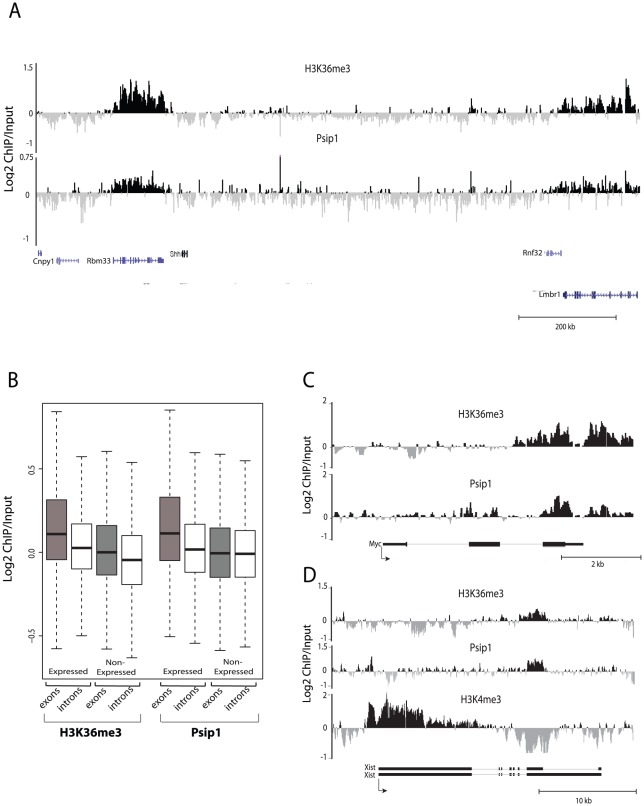
Genomic distribution of Psip1/p52 and H3K36me3. A) Mean log2 ChIP∶input for Psip1/p52 and H3K36me3 in MEFs for an approximately 1.2Mb genomic window from mouse chromosome 5. n = 4 (3 biological and 1 technical replicate). B) Box plots showing the distribution of log2 ChIP∶input for Psip1/p52 and H3K36me3 across exons and introns of expressed or non-expressed genes. Data are deposited in NCBI GEO (Accession no. GSM697402-GSM697411). C, D) Mean log2 ChIP∶input for Psip1/p52 and H3K36me3 in MEFs at (C) c-*Myc* and (D) *Xist* loci. H3K4me3 is also shown for XIST. Filled boxes indicate the positions of exons. n = 4 (3 biological and 1 technical replicate) for H3K36me3 and Psip1. NCBI GEO accession number for array platform is GPL13276. n = 2 biological: replicates for H3K4me3.

The large-scale distributions of H3K36me3 and Psip1/p52 were similar to each other and both appeared to be enriched at active genes (Figure 2A). Across the entire array, levels of both Psip1/p52 and H3K36me3 were significantly higher at active genes than inactive genes or intergenic regions, and furthermore were especially enriched at the exons compared to the introns of expressed genes (p<0.05) (Figure 2B). Visual inspection of specific genes revealed a similar distribution of Psip1/p52 and H3K36me3 at some downstream exons (Figure 2C and 2D), distinct from the peak of H3K4me3 at promoters. However, there is also evidence for some enrichment of Psip1/p52 near the transcription start sites (TSSs) suggesting multiple modes of Psip1 association to chromatin. Correlation between the distribution of Psip1/p52 and H3K36me3 (Spearman's rank correlation coefficient ρ = 0.38, p<0.05) was stronger than that between Psip1/p52 and H3K4me3 (ρ = −0.05) or between H3K36me3 and H3K4me3 (ρ = 0.013).

### Splicing proteins interact with Psip1/p52

To determine whether there are other interacting partners for Psip1 isoforms, apart from H3K36me3, we performed immunoprecipitation with two different antibodies.

**Figure 3:**
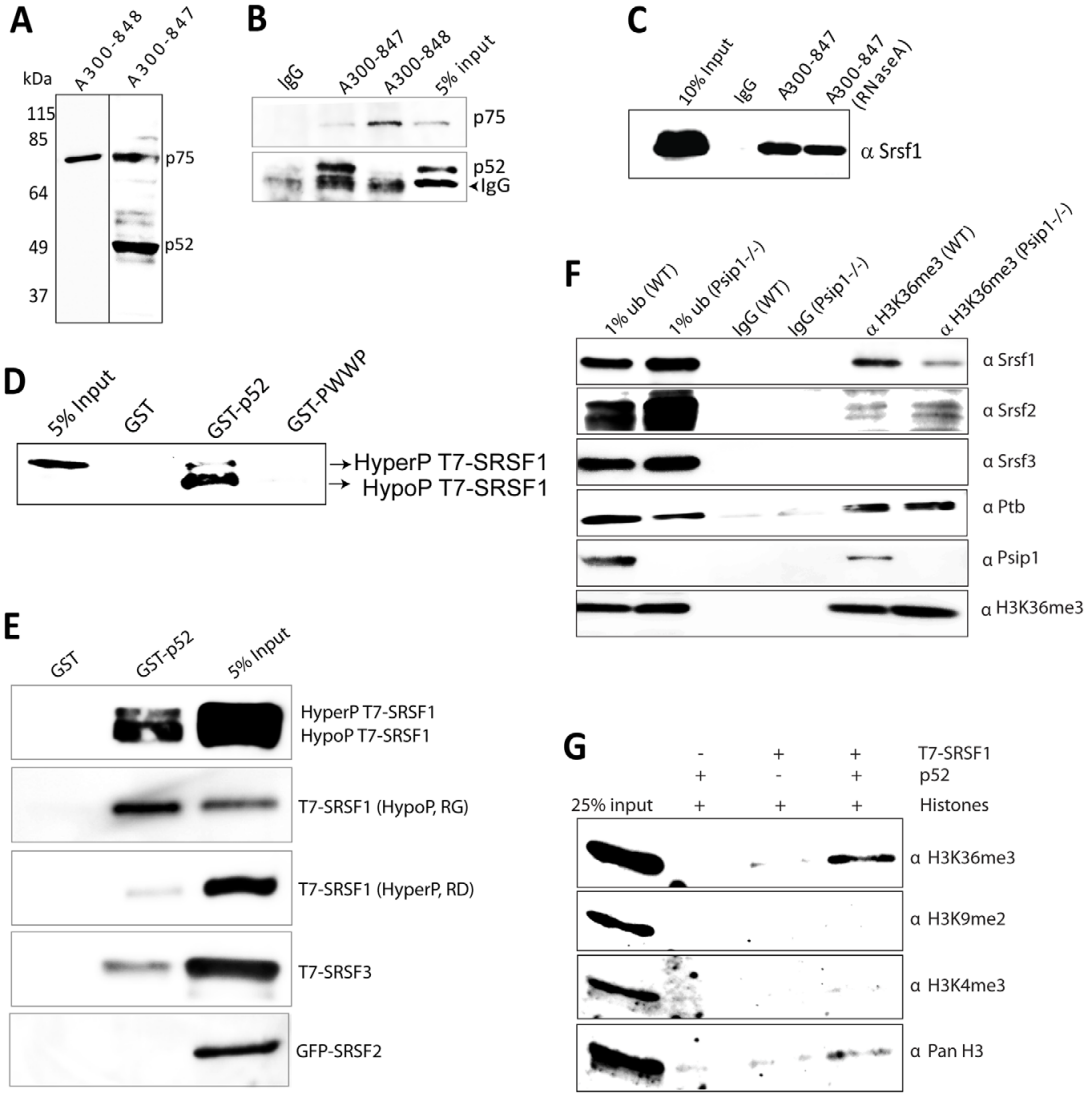
Immunoprecipitation of Psip1/p52 and p75. A) Immunoblot of NIH 3T3 nuclear extract with antibodies; A300-848 which recognizes only the p75 isoform of Psip1, and A300-847 which detects both p52 and p75. B) IPs with IgG, A300-847 and A300-848 from NIH 3T3 nuclear extracts, immunoblotted with antibodies recognizing p75 (A300-848) or p52 (A300-847). Input is 5% total extract. C) Immunoblot of A300-847 IPs with αSRSF1. IP was also performed in the presence of RNase A. Input is 10% of total extract and IgG served as a control. D) In vitro pulldown of 293T cell expressed T7-SRSF1 using GST-p52 and Psip1-PWWP and immunoblotted with αT7. Input is 5% of T7-SRSF1 and GST alone is control. E) In vitro pulldown with GST-p52 of; T7-SRSF1 and mutants that mimic its hypo-(RG) and hyper-phosporylation (RD), T7-SRSF3 and GFP-SRSF2. Immunoblotting was with αT7 or αGFP. F) ChIP with αH3K36me3 from wild-type (wt) and *Psip1* ^−/−^ MEFs immunoblotted with antibodies detecting Srsf1, Srsf2, Srsf3, PTB, Psip1 and H3K36me3. G) In vitro pulldown of HeLa core histones by T7-SRSF1 in the presence or absence of Psip1/p52 and immunoblotted with antibodies detecting pan H3, H3K36me3, H3K9me2 and H3K4me3.

Antibody A300-847 was raised against an epitope present in both p52 and p75 (a.a. 225–275) (Figure 1A) and indeed detects both isoforms by immunoblot (Figure 3A). However, A300-847 efficiently IPs the Psip1 p52 isoform, but not p75 (Figure 3B). This is likely due to masking of the A300-847 epitope in the p75 tertiary structure. In agreement with this, Ge et al [38] also reported that antibodies generated against recombinant p52 could recognize both p52 and p75 by immunoblot, but could not IP Psip1/p75 under native conditions.

In addition to Psip1/p52 itself, a large number of other proteins were co-immunoprecipitated from NIH3T3 cells using A300-847 (Figure S1A). Mass spectrometry revealed that ≈95% of them are known to function in pre-mRNA processing. Grouping the mascot hits according to their known function(s) and/or key domains revealed; SR proteins, DEAD/H box helicases, proteins of the U5 snRNP, hnRNP proteins, and other proteins known to function in pre-mRNA processing (Table 1). Apart from these, a few other transcription related proteins were identified. In agreement with the report of its co-purification with p52, Srsf1 was one of the major hits [38]. The specificity of A300-847 antibody for wild-type (wt) Psip1/p52 is evidenced by the absence of immunoprecipitation of Srsf1 and other SR proteins in extracts prepared from MEFs homozygous for a gene-trap integration into Psip1 (*Psip1^gt/gt^*) (Figure S1) in which the A300-847 epitope is 3′ to the site of gene trap integration, and so is absent from the resulting fusion protein (Figure 1A) [22].

**Table 1:** Psip1/p52 interacting partners.

ID	Protein name	Alias	Protein domains	Functions	Peptides	Mass (kDa)
**SR proteins**						
gi 5902076	Srsf1 *	SF2/ASF	RRM x2, RS	Constitutive and AS	42	27
gi 3153821	Srrm 1 *	POP101/Srm160	RS, PWI	Constitutive and AS	30	101
gi 22122585	Srsf7	9G8	RRM, RS	Constitutive and AS	25	27
gi 6755478	Srsf2	SC-35 *	RRM, RS	Constitutive and AS	25	35
gi 3929369	Srsf5	SRp40	RRM x2, RS	Constitutive and AS	17	31
gi 114152161	Srrm2	SRm300	RS	Constitutive and AS	17	295
gi 9622185	Acinus	Acin1	RRM, RS, SAP	mRNA splicing regulation, apoptosis, EJC	16	150
gi 12844972	Srsf 3	Srp20	RRM, RS	Constitutive and AS	16	19
gi 27461725	Pinin	Pnn	RS	AS regulator	11	82
gi 4759098	Srsf10*	Tra2b	RRM, RS x2	Splicing activator	9	33
gi 13278367	Rbm39	Rnpc2	RRM x3, RS	AS regulation	9	47
gi 13385016	Srsf9	SRp30	RRM x2, RS	Constitutive and AS	8	25
gi 124487333	Ppig	SRCyp	RS, PPIase	Regulates localisation of SR proteins	6	88
gi 1399464	Prpf4b	Prpk	RS, kinase	mRNA splicing	6	116
gi 4001720	SRp38	Nssr 1	RRM, RS	General splicing repressor	3	31
gi 122890231	Srsf4	Srp75	RRM x2, RS	Constitutive and AS	2	56
**DExD/H box helicases**						
gi 28076989	DDX54	DP97	DEAD, helicase	Transcription regulation	43	98
gi 148707489	DHX 9	NDH2	DsRM, DEAD, helicase	Transcription regulation, mRNA splicing	30	132
gi 2500527	DDX5 *	RNA helicase p68	DEAD, helicase	mRNA splicing, microRNA processing	27	69
gi 6753620	DDX3X	DAP88	DEAD, helicase	mRNA splicing, RNA export, microRNA processing, translation	27	73
gi 16716475	DDX 50	RH II/Gu	DEAD, helicase	Ribosome biogenesis, transcription co-factor	25	82
gi 27371129	DDX10	HRH-J8	DEAD, helicase	Ribosome biogenesis	24	78
gi 110835726	DHX15	Prp43	DEAD, helicase	mRNA splicing, microRNA processing	27	73
gi 73620772	DDX41*	Abstrakt	DEAD, helicase, C2HC Zn finger	mRNA splicing	19	70
gi 25141235	DDX3Y	DBY	DEAD Box	mRNA splicing, microRNA processing, RNA export, translation	14	73
**SnRNPs**						
gi 40018610	U5-200KD	BRR2	DEAD, helicase, sec63	mRNA splicing, U5 snRNP complex	13	246
gi 124430514	U5 100K *	DDX 23/Prp28	RS, DEAD, helicase	mRNA splicing, U5 snRNP complex	12	42
gi 19527174	SF3B3*	Sf3b(130)	CPSF_A	mRNA splicing, U2 snRNP complex	10	136
gi 21539655	U5-102 kDa	Prp6	HAT repeats	mRNA splicing, U5 snRNP complex	6	107
**hnRNPs**						
gi 21313308	hnRNPm	KIAA4193	RRM	Pre-mRNA processing	17	78
gi 3914804	hnRNPg	Rbmx	RRM	Pre-mRNA processing	10	42
gi 123778087	hnRNPu like	MLF1ANP	RRM, SAR DNA binding	Pre-mRNA processing	4	85
gi 13384620	hnRNPk	TUNP	KH domains	Pre-mRNA process	5	51
**Other proteins likely to be involved in RNA processing**						
gi 54607128	RRP5	PDCD11	S1, HAT	rRNA processing	98	209
gi 29747798	Rbm 12b	RBM 12Ba	RRM x5	Unknown	12	97
gi 188497644	Noc3	Fad24		mRNA splicing, transcription	11	93
gi 83921589	Fxr2		KH		10	74
gi 4835742	FXR1	Fxr1p	KH	translation	8	64
gi 29747807	Safb2 *	SAF-B2	SAP, RRM	Transcription and splicing	4	114
gi 34098365	NRF	ITBA4	ds RNA binding, G patch	Transcription regulation	11	78
**Other proteins**						
gi 23503105	NOC2L			nucleolar transcription corepressor	30	86
gi 18539461	NOL6			nucleolar	30	128
gi 2645205	Mybbp1A		armadillo	Transcription regulation	28	150
gi 9790013	AATF			Nucleolus, apoptosis, transcription regulation	25	59
gi 71979675	LAS1L			Ribosome biogenesis	23	88
gi 83921589	FMR2				10	74

Proteins identified by mass spectrometry of p52 IPs (200 mM KCl).

*: indicates known proteins of the ‘spliceosomal complex C’. Data on protein domains and putative protein functions were taken from http://npd.hgu.mrc.ac.uk/.

Antibody A300-848 specifically recognizes the extreme C-terminus – amino acids (a.a.) 480 to 530 - of Psip1/p75 (Figure 1A) and so detects endogenous p75, but not p52, in immunoblots and IPs (Figure 3A and 3B). Only a few transcription related proteins, in addition to p75 itself, were IP'ed from nuclear extracts by A300-848 (data not shown).

These data indicate a cellular link between Psip1/p52 and the splicing machinery. Immunoblotting of the IP from RNase treated nuclear extracts indicated that Psip1/p52 interacts mainly with the hypophosphorylated form of SRSF1 (Figure 3C). Phosphorylation levels of SR proteins are known to modulate alternative splicing and alter SR protein distribution in relative to splicing-factor enriched nuclear speckles [46]–[48]. GST-p52 pull down of T7-SRSF1 (over expressed HEK-293T cells), confirmed direct interaction of Psip1/p52 with SRSF1 and that the Psip1 PWWP domain is not sufficient for this (Figure 3D). Furthermore, GST-p52 pulldown of SRSF1 mutants which mimic hypo (RG) and hyper (RD) phosphorylation (serine residues within RS/SR dipeptide repeats of RS domain substituted with Glycine: RG or Aspartic acid: RD) [49] shows higher affinity of Psip1/p52 for hypophosphorylated SRSF1 compared to the hyperphosphorylated form (Figure 3E).

GST pulldown also confirms interaction with SRSF3, but shows that Psip1/p52 does not simply interact non-specifically with all SR proteins, since there is no direct interaction with SRSF2 (SC35) (Figure 3E). Identification of Srsf2 by mass spectrometry in the A300-847 immunoprecipitate presumably is the result of indirect association with other splicing proteins (Table 1).

### Association of SRSF1 to H3K36me3 marked chromatin requires p52

Unphosphorylated SRSF1 has been reported to associate with chromatin, especially the H3 tail and to be sensitive to H3 tail post-translational modifications [50]. To investigate whether absence of Psip1 causes any loss of Srsf1 chromatin association in vivo, chromatin purified by ChIP for H3K36me3 was analyzed by immunoblotting. Levels of Srsf1 associated with H3K36me3 modified chromatin were greatly reduced in Psip1^−/−^ MEFs cells that do not have detectable Psip1/Ledgf [25], compared to wild type (Figure 3F). As controls, the levels of H3K36me3 associated Ptb and Srsf2 were not changed in the Psip1^−/−^ IPed chromatin compared to wild type, These results confirm that Psip1/p52 specifically recruits Srsf1 to H3K36me3 chromatin in vivo, but not Ptb, which has been shown to be recruited to H3K36me3 chromatin through MRG-15 [20].

To investigate whether SRSF1 alone can bind to H3K36me3 in vitro, or whether this occurs via interaction with Psip1, we pulled-down HeLa core histones with T7-SRSF1, with or without addition of Psip1/p52. Immunoblotting with antibodies recognizing different methylated states of H3 revealed a specific enrichment of H3K36me3 in the presence of Psip1/p52 compared to SRSF1 alone (Figure 3G). These results suggest that Psip1/p52 can aid the recruitment of specific splicing factors, including SRSF1, to H3K36me3 modified chromatin.

### Psip1 p52 co-localizes with splicing factors

**Figure 4:**
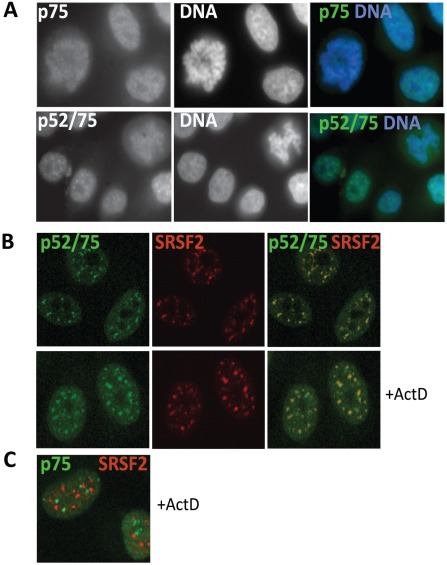
Sub-cellular localization of Psip1/p52 and p75. A) Immunofluorescence and wide-field epifluorescence microscopy on human cells with; (upper row) p75-specific antibody A300-848, (lower row) A300-847 which can recognize both p52 and p75. DNA was counterstained with DAPI. B) Co-immunofluorescence of Psip1/p52 (green/A300-847) and SRSF2 (red) analyzed by confocal microscopy in untreated (upper row), or actinomycin D (ActD) treated cells. C) Co-immunofluorescence of Psip1/p75 (green/A300-848) and SRSF2 (red) in ActD treated cells and analyzed by confocal microscopy.

Given the preponderance of splicing/RNA-binding proteins co-immunoprecipitating with Psip1/p52 but not p75 (Figure 3 and Table 1), we investigated the nuclear localizations of Psip1 isoforms. Antibody A300-848 revealed that, as for *Psip1^gt/gt^* [22], endogenous p75 is associated with chromosomes in mitotic cells (Figure 4A) and is generally distributed in the nucleoplasm at interphase.

Immunostaining with A300-847 also showed association with mitotic chromosomes, but at interphase revealed numerous nuclear foci reminiscent of splicing-factor enriched nuclear speckles [51] (Figure 4A). Co-immunostaining for Psip1/p52/p75 and SRSF2, a marker for the splicing-factor enriched nuclear speckles, confirmed this (Figure 4B). Splicing-factor enriched nuclear speckles become larger and less numerous upon the inhibition of transcription with actinomycin D [40]. Concomitantly, Psip1/p52 also became redistributed to these foci. In contrast, there was no correspondence between the sub-nuclear distribution of Psip1/p75 and splicing-factor enriched nuclear speckles (Figure 4C).

### Loss of Psip1/p52 affects alternative splicing

To identify whether there are specific exons whose splicing in vivo might be affected by Psip1/p52, we analyzed patterns of alternative splicing in RNA prepared from primary MEFs from three different *Psip1* ^gt/gt^ and corresponding wild type littermate embryos. *Psip1* ^gt/gt^ mutant mice were generated from ES cells with a gene trap integrated between exons 8 and 9 of *Psip1*. This results in the production of a protein in which only the N-terminal 208 a.a. of Psip1 are present (arrowed in Figure 1A) and are fused to the β-geo reporter [40]. We used a custom Affymetrix microarray containing 40,443 exon junction probe sets derived from 7,175 genes with one or more predicted alternative transcripts and analyzed the data with ASPIRE 3 software [52]. Splicing changes were detected in 95 alternative exons with a score that, in our past experience, can be validated by RT-PCR with high (>90% success; ΔI rank ≥1, or ≤−1) [53], [54]. Out of these, 58 exons, from 55 genes, appeared to have decreased inclusion in the mutant MEFs and 37 exons, from 35 genes, had increased inclusion (Table S2).

**Figure 5:**
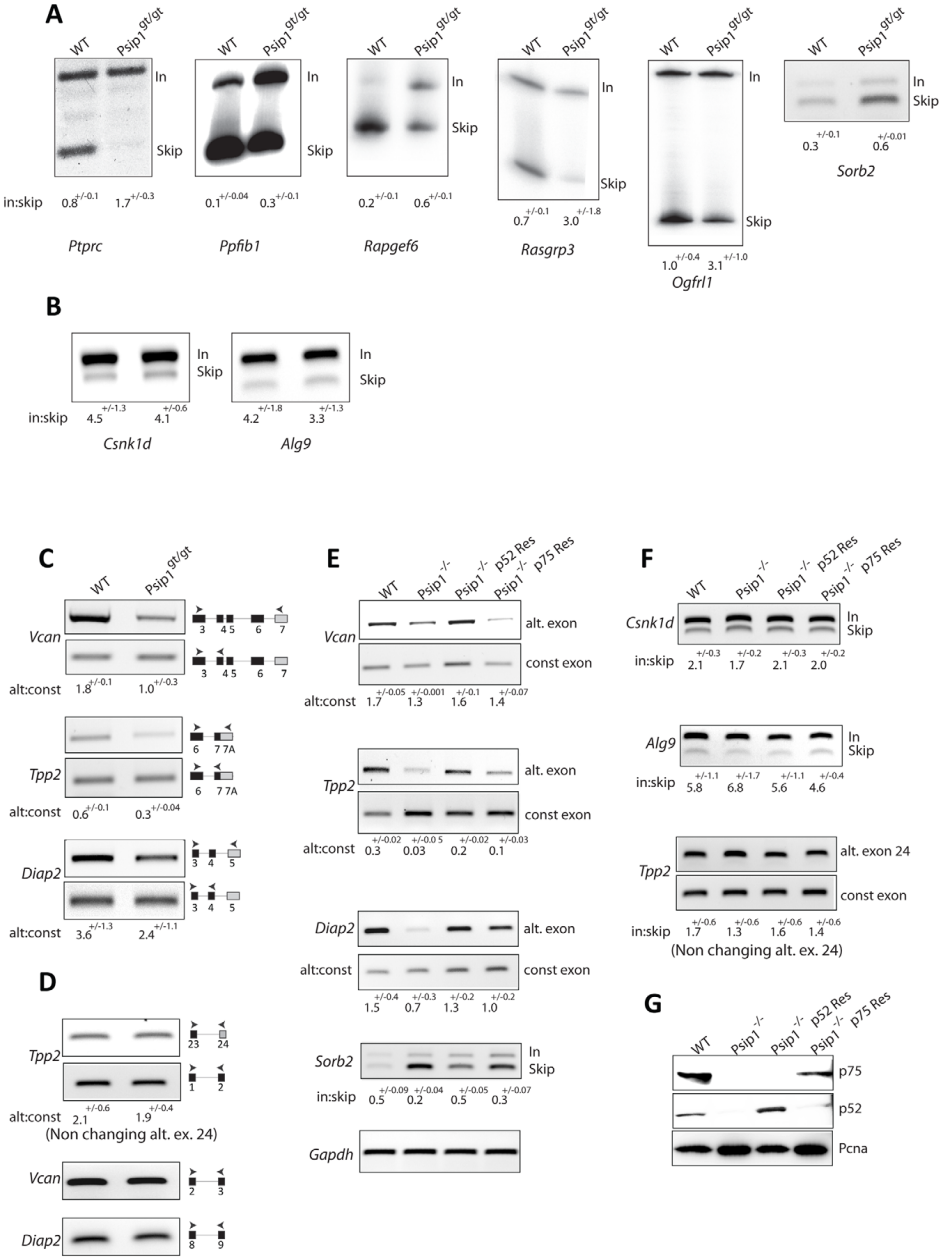
Alternative splicing in *Psip1* ^gt/gt^ cells. RT-PCR to detect; exon inclusion (In) or skipping (Skip) of (A); *Ptprc*, *Ppfibp1*, *Rapgef6*, *Rasgrp3*, *Ogfrl*, and *Sorb2* all of which showed evidence for altered alternative splicing in array analysis, or (B) *Csnk1d and Alg9*, *which were unchanged in the array*, in RNAs prepared from wt and *Psip1* ^gt/gt^ primary MEFs. C) Specific exon-exon junctions (constitutive-constitutive or constitutive-alternative) of *Vcan*, *Tpp2* and *Diap2* in wt and *Psip1* ^gt/gt^ MEFs. D) Specific exon-exon junctions (constitutive-constitutive) of *Vcan* (5′ exons) and *Diap2* (3′exons) and constitutive-constitutive (5′) and constitutive-alternative of *Tpp2* alternative exon 24 in wt and *Psip1* ^gt/gt^ MEFs. E) Specific exon-exon junctions (constitutive-constitutive or constitutive-alternative) of *Vcan*, *Tpp2 and Diap2*; exon inclusion (In) or skipping (Skip) of *Sorb2* in wt and *Psip1* ^−/−^ MEFs, and after transfection of p52 or p75 Psip1 into *Psip1* ^−/−^ MEFs. F) Exon inclusion (In) or skipping (Skip) of; *Csnk1d* Alg9, and constitutive-constitutive (5′) and constitutive-alternative of *Tpp2* alternative exon 24 in RNAs prepared from wt and *Psip1* ^−/−^ MEFs, and after transfection of p52 or p75 Psip1 into *Psip1* ^−/−^ MEFs. Sequence and position of primer pairs for each exons are listed in Table S3. Below the gels in panels A to F, the mean ratio of alternative∶constitutive exon (+/− s.e.m.) is shown for three biological replicates. G) Immunoblots of proteins using A300-847 antibodies to detect p75 and p52 in wt and *Psip1* ^−/−^ MEFs, also *Psip1* ^−/−^ MEFs transfected with p52 (*Psip1* ^−/−^ p52Res) and p75 (*Psip1* ^−/−^ p75Res). Immunoblot with Pcna served as a loading control.

The gene-trap in *Psip1* ^gt/gt^ is between exons 8 and 9 (Figure 1A) [22] so the resulting mRNA lacks exons 9-15. This was evident from the microarray results, which detected *Psip1* exons 11 and 12 as those with the most decreased inclusion in the whole analysis (Table S2). At the other extreme, the most increased inclusion of alternative exons in *Psip1* ^gt/gt^ was at *Ptprc*. In mutant cells, increased alternative exon inclusion for *Ptprc*, *Ppfibp*, *Rapgef6*, *Rasgrp3* and *Ogfrl1*, all of which have a ΔI>1, and altered 3′ splice site utilization at alternative exon 4 of *Sorb2* (ΔI of <−1), was confirmed by semi-quantitative RT-PCR of RNA from primary MEFs derived from three wild type and three *Psip1* ^gt/gt^ litter mates (Figure 5A). Primer pairs spanned across regions subject to alternative splicing to generate PCR products of different sizes dependent on exon skipping or inclusion (Table S3). A 2–3 fold increase in the ratio of included∶skipped exon bands was seen in mutant cells compared to wild-type. The absence of alternative splicing at the alternative exons of *Csnk1d*, *Alg9* and *Tpp2* exon 24, which were not detectably altered in the microarray, was also confirmed by RT-PCR (Figure 5B, 5D).

To examine the splicing of specific alternative exons, RT-PCR was also carried out across specific constitutive exon - constitutive exon junctions and across constitutive exon - alternative exon junctions of *Vcan*, *Tpp2* and *Diap2* where microarray analysis had indicated increased exon skipping in *Psip1^gt/gt^* cells (ΔI≤−1) (Table S2). This confirmed the decreased inclusion of alternatively spliced exons in *Psip^gt/gt^* cells (Figure 5C). To rule out the possibility of amplification bias, RT-PCR using primers spanning constitutive exons at either the 5′ or 3′ end of *Tpp2*, *Vcan* and *Diap2* were tested (Figure 5D).

Although the gene-trapped Psip1 protein produced in *Psip^gt/gt^* cells is truncated and co-localizes with concentrations of chromatin instead of splicing factors [22], [40], we wished to confirm a role for Psip1 in the regulation of alternative splicing using an independently derived mutant allele. Therefore, splicing patterns of specific genes were also examined in *Psip1* ^−/−^ MEFs in which deletion of *Psip1* exon 3 leads to the absence of detectable Psip1/Ledgf protein [25]. As for *Psip1^gt/gt^* (Figure 5A and 5B) altered patterns of splicing at *Vcan, Tpp2,Diap2 and Sorb2* were detected in RNA prepared from *Psip1* ^−/−^ MEFs compared to wild-type controls (Figure 5E).

Since the mutations in both *Psip1* ^gt/gt^ and *Psip1* ^−/−^ affect both p52 and p75 isoforms, we determined whether dysregulated alternative splicing could be directly attributed to p52 rather than p75 by complementing *Psip1* ^−/−^ MEFs with expression of either p52 and p75 (Figure 5G). Only expression of p52 rescued the changes in alternative splicing pattern in *Psip1* ^−/−^ cells. Expression of Psip1/p75 did not restore splicing patterns of the tested genes (Figure 5E). Consistent with the microarray, RT-PCR of alternative exons of *Csnk1d*, *Alg9* and alternative exon 24 of *Tpp2* were not significantly altered by loss of Psip1 (*Psip1* ^−/−^) or by functional rescue of those cells with either p52 or p75 (Figure 5F).

### Loss of Psip1/p52 alters Srsf1 localization

**Figure 6:**
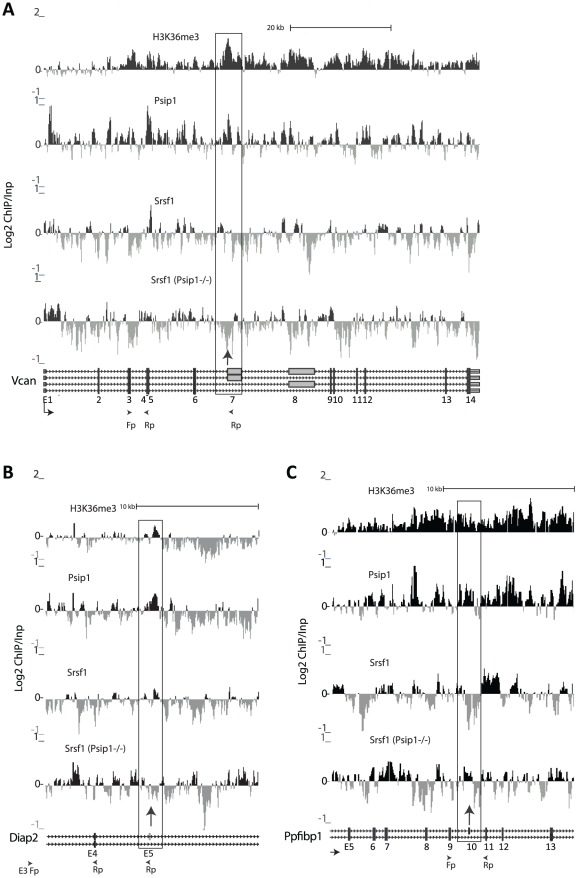
ChIP for H3K36me3, Psip1, and Srsf1 in wt and Psip1 mutant MEFs. A) Mean log2 ChIP∶input for H3K36me3, Psip1 and Srsf1 in wt MEFs across the *Vcan* (A), *Diap2* (B) and *Ppfibp1* (C) loci. Distribution of Srsf1 in chromatin from *Psip1* ^−/−^ MEFs is also shown. Filled boxes indicate the positions of exons and the arrows indicate the position of alternatively spliced exons whose inclusion into spliced mRNAs is altered in *Psip1* ^gt/gt^ cells. n = 2 biological replicates that also incorporate a technical (dye-swap) replicate. Array platform number is GPL14175 and the GEO accession numbers for ChIP data are; GSM782590 (Psip1), GSM782591 (H3K36me3), GSM782592 and GSM782593 (Srsf1 in wt), GSM782594 and GSM782595 (Srsf1 in *Psip1* ^−/−^).

Our data suggest that the absence of Psip1/p52 alters the splicing pattern of alternative exons and that this might be mediated by perturbed association of splicing factors at specific genomic loci. SR proteins such as SRSF1 can affect alternative splicing patterns through their recruitment to both alternatively and constitutively spliced exons [55]. Therefore, we examined the enrichment of H3K36me3, Psip1/p52, and Srsf1 across some gene loci subject to alternative splicing, by ChIP and hybridization to a custom microarray encompassing 8.2 megabases of the mouse genome including loci whose splicing pattern we have shown (Figure 5) is altered in *Psip1* ^gt/gt^ cells. In addition Srsf1 binding was analyzed by ChIP from cells lacking Psip1 (*Psip1* ^−/−^ MEFs) (Figure 6). The correlation between sites of Srsf1 localization and the Psip1 bound sites in wild-type cells (ρ = 0.35 p<0.05), was reduced (ρ = 0.25) in *Psip1^−/−^* cells. In *Psip1* ^−/−^ cells Srsf1 binding was lost from the 5′ side of *Vcan* exon 7 (Figure 6A), whose inclusion into processed mRNA is reduced in *Psip1* ^gt/gt^ cells (Figure 5). Similarly, at *Diap2* Srsf1 binding in *Psip1* ^−/−^ cells was lost to the 3′ side of exon 5 (Figure 6B) whose inclusion is reduced in *Psip1* ^gt/gt^ cells (Figure 5B).

However, the affects of Psip1 loss on Srsf1 chromatin binding are complex. At *Ppfibp1*, where there is increased alternative exon inclusion in *Psip1* ^gt/gt^ cells (Figure 5), sites of Srsf1 binding seems displaced toward the alternatively spliced exon 10, and away from the downstream constitutively spliced exon 11 in mutant cells (Figure 6C). This likely reflects a shift in the balance between different modes of Srsf1 recruitment across this locus in the absence of Psip1.

## Discussion

Tri-methylation of H3K36 is elevated in the expressed exons compared to introns, which suggested it is linked to splicing. A recent report showed specific recruitment of the splicing factor PTB to H3K36me3 modified chromatin at the *FGFR2* gene via MRG15 [20]. It was not clear whether other similar proteins exist to recruit different splicing factors to H3K36me3 modified nucleosomes. Our results suggest that there is a more extensive family of chromatin proteins which can bind to H3K36me3 and also recruit splicing factors to facilitate alternative splicing. However, recent investigations [18], [19] also propose a plausible but not mutually exclusive model, in which splicing modulates the level of H3K36me3. This suggests that there is extensive interplay between H3K36me3 chromatin modification and alternative splicing.

We demonstrate that the short (p52) isoform of Psip1 modulates the inclusion or exclusion of alternative exons in specific mRNAs, probably by interacting both with chromatin and proteins involved in pre-mRNA splicing. Despite containing almost all the a.a. residues of p52, the longer (p75) Psip1 isoform neither co-IPs, nor co-localizes, with splicing related proteins (Figure 4). This, together with the inability of the A300-847 antibody to IP p75, even though its epitope is present in the protein sequence and recognized in denatured p75 by immunoblot (Figure 3), suggests that protein folding of Psip1/p75 occludes both the A300-847 epitope and the region capable of interaction with splicing factors. Differential localization and interaction with the transcriptional regulation machinery or with splicing proteins has previously been reported for different isoforms of another protein – WT1 [56]–[58].

We add Psip1 to the recently identified group of PWWP-containing proteins - Brpf1, Dnmt3a, MSH-6, NSD1, NSD2 and N-PAC - that have been shown to be able to bind H3K36me3 [43]–[45] (Figure 1 and Figure 2). This establishes the PWWP members of the ‘royal’ family of protein domains as reader of this histone modification, that has been associated with the exons of active genes [13]–[16], [59] and whose deposition onto chromatin has recently been linked to the process of splicing itself [18], [19]. MRG15 uses a chromo-domain for methylated histone binding [20]. The chromo-domain, like PWWP, is also a royal family protein domain [42], but the chromo-domain of MRG15 is structurally more similar to the PWWP domain of DNMT3b than to that of more typical chromo-domain proteins that recognize H3K9me3 or H3K27me3 [60].

Psip1/p75 was demonstrated to be important for guiding HIV/lentiviral integration to the body of genes [24]–[26]. Our demonstration that the N-terminal PWWP domain, shared by both p52 and p75 Psip1 isoforms, recognizes and binds to H3K36me3 provides a mechanistic explanation for this pattern of HIV integration.

There is a growing awareness of the interactions between splicing factors and RNA polymerase II elongation [61] and emerging evidence now highlights the role of histone modifications in this process. At gene promoters, the chromo-domains of CHD1 recognize H3K4me3 [62], [63] and CHD1 interacts with the SF3a subcomplex of the U2 snRNP to then facilitate mRNA splicing post-initiation [7]. Similarly, in yeast, the histone acetyltransferase GCN5, found at the promoter regions of active genes, also interacts with components of the U2 snRNP [5]. MRG15 and Psip1/p52 now provide two examples of H3K36me3 binding proteins that can influence the recruitment of splicing components to chromatin.

MRG15 interacts with the RNA–binding protein PTB to regulate alternative splicing [20]. In contrast, we found interactions between Psip1/p52 and; several SR-containing proteins – including Srsf1 (Figure 3 and Table 1), components of the U5 snRNP and other proteins involved in RNA processing. Furthermore, we show that the absence of functional p52 affects alternative splicing of defined endogenous genes in vivo (Figure 5) and alters the pattern of Srsf1 binding across alternatively spliced gene loci (Figure 6).

Differential expression of SR proteins is important for tissue-specific alternative splicing and is abundant in brain and testis [64], [65] where, compared to other tissues, mRNA for the p52 isoform of Psip1 is also at high levels compared to that of p75 [21].

Amongst other Psip1 co-immunoprecipitating proteins are many DExD/H box family putative RNA helicases. One of these is DDX10 which, like Psip1/Ledgf, is found as a fusion partner with Nup98 in myeloid leukaemias and myelodysplastic syndromes [66]–[69], perhaps indicative of their function in a common pathway that is mis-regulated in these malignancies. The presence of the H3K36 methyltransferase NSD1 as another Nup98 fusion partner [70] [71] suggests that the splicing-H3K36me3 connection might be implicated in the aetiology of these myeloid disorders.

## Materials and Methods

### Histone tail peptides arrays and peptide pulldown

A modified histone peptide array (Active motif, #13005) was blocked in TBST buffer (10 mM Tris/HCl pH 8.3, 0.05% Tween-20, 150 mM NaCl) containing 5% non-fat dried milk at 4°C overnight. The membrane was washed with TBST for 5 min, and incubated with 10 ηM purified GST-tagged Psip1 PWWP domain, or GST protein alone, at room temperature (rt) for 1 h in interaction buffer (100 mM KCl, 20 mM HEPES pH 7.5, 1 mM EDTA, 0.1 mM DTT, 10% glycerol). After washing in TBST, the membrane was incubated with goat α-GST (GE Healthcare #27- 4577-01, 1∶5000 dilution in TBST) for 1 h at rt. The membrane was then washed 3× with TBST for 10 min each at rt and incubated with horseradish peroxidase conjugated α Goat antibody (Invitrogen #81-1620 1∶12000 in TBST) for 1 h at rt. The membrane was submerged in ECL developing solution (Pierce, #32209), imaged (Image-quant, GE Healthcare) and the data quantified using array analyzer software (Active motif).

Biotinylated histone H3 (Ana spec 64440-025) and H3K36me3 (Ana spec 64441-025) peptides coupled to Streptavidin magnetic beads (Invitrogen 656-01), and were used to pull-down GST-p52 as described (http://www.epigenome-noe.net/WWW/researchtools/protocol.php?protid=46).

### Cloning, expression, and purification of proteins

Mouse GST-p52 and GST-PWWP (a.a. 1–97), were cloned into pDEST-PGEX6P. Proteins were expressed in BL21 Codonplus *E.coli* and purified on glutathione sepharose using standard protocols.

Human SRSF1 and Human Psip1/p52 open reading frames were cloned into pCG-T7 and pEGFP vector with CMV promoters. pIRES2-eGFP-p52-HA and pIRES2-eGFP-p75-HA were kindly gifted by Prof. Alan Engelman (Dana-Farber Cancer Institute).

### Immunoblotting

Immunoblotting was performed with the following antibody dilutions A300 847(1∶2000), A300-848 (1;3000), αH3K36me3 (Abcam AB9050,1∶500). αH3K9me2 (Abcam ab7312,1∶500) αH3K4me3 (Millipore 07-473, 1∶500), αPan H3 (Abcam Ab 1791) αSRSF1 (1∶300), αSRSF1 (Invitrogen 32-4500 1∶2000) αPCNA (Santa Cruz, Sc56) αT7 (Novagen, 65922), Detection was by ECL.

### Cell culture and transfection

Mouse embryonic fibroblast (MEF) lines were derived from 13.5 day old *Psip1^gt/gt^* embryos and their corresponding wild-type littermates [22]. They were maintained for three passages in DMEM supplemented with 15% Fetal calf serum (FCS), non-essential amino acids, sodium pyruvate, L-glutamine, and Penicillin/Streptomycin and cultured at 37°C.

Psip1^−/−^ and corresponding wild-type MEFs (gift of Alan Engelman) [25] were maintained in DMEM supplemented with 10% FCS and Pencillin/Streptomycin. They were transfected with Lipofectamine and GFP^+ve^ FACS-sorted cells were harvested after 72 hrs.

### Chromatin immunoprecipitation

MEFs were harvested by trypsinizing and fixed immediately with 1% formaldehyde (25°C, 10 min) in PBS, and stopped with 0.125M Glycine. Chromatin immunoprecipitation (ChiP) was performed as described previously [72]. Nuclei were sonicated using a Diagenode Bioruptor (Liege, full power 30 s on, 30 s off, in an icebath for 50 min) to produce fragments of <300 bp. An arbitrary concentration of 200 µg chromatin was incubated with 4 µg rabbit IgG (Santa Cruz, sc-2025), Psip1 antibodies (A300-847), H3K36me3 antibodies (Abcam, Ab 9050-100), αH3K4me3 (Millipore 07-473) or αSRSF1 (Invitrogen 32-4500) and washed, eluted and cross-links reversed.

### Histone association assay

To analyze proteins associated with H3K36me3, αH3K36me3 ChIP'ed chromatin was heated at 95 C in the presence of 1× Laemmli buffer for 10 min, separated on 4–20% SDS-PAGE, transferred onto a PVDF membrane, and probed with αSRSF1, α SRSF2, αSRSF3, αPTB (Invitrogen 32-4800) αPsip1 (A300-847A), and αH3K36me3 antibodies. Instead of species-specific secondary antibodies, HRP coupled Clean-Blot IP Detection Reagent (Thermo Scientific Prod. No. 21230) was used to avoid cross reactivity of HRP coupled antibody to denatured IgGs in the gel.

### ChiP on chip for Psip1, H3K36me3, H3K4me3, and Srsf1

For analysis in Figure 2, WGA2 amplified ChIP DNA and input DNA were labeled and hybridized according to the manufacturer's protocol to a 3×720,000 probe custom microarray containing specific tiled regions encompassing 8.2 megabases of the mouse genome (Nimblegen). Array platform number is GPL13276 and the GEO accession numbers for ChIP data are; Psip1: GSM697402, GSM697403, GSM697404, GSM697405, H3K36me3: GSM697406, GSM697407, GSM697408, GSM697409, H3K4me3: GSM697410- GSM697411.

Biological replicates were performed for all the ChIP array experiments and the data were analyzed in R/Bioconductor (http://genomebiology.com/2004/5/10/R80) using the Epigenome (PROT43) protocol (http://www.epigenome-noe.net/WWW/researchtools/protocol.php?protid=43) with the following parameters; The mean signal intensity of the 4 replicate probes present on each array was calculated. Loess normalization was used within arrays to correct for dye bias, and scale normalization was used within replicate groups to control inter-array variability. Log enrichment for each group was calculated by subtracting the mean log2 input intensities from the mean of log2 ChIP-enriched intensities. Probes were tested for significant enrichment using the significance analysis of microarrays (SAM) technique [73], and the local false discovery rate based on the SAM statistic was calculated using the Locfdr R package [74]. A false discovery rate of 0.05 was used as the significance cutoff. The spearman rank correlation coefficient was used to assess the correlation between replicate experiments.

The spearman rank correlation coefficient was used on all log enrichment scores between data from Psip1 ChIP and remaining groups to determine, significance and strength of their relationship.

To determine if overlaps between Psip1, H3K36me3 and H3K4me3 enriched probes were significant, 1000 randomized datasets were produced and the 95th percentile of the resultant overlaps was used as a significance cutoff.

To determine the enrichment of probes over genomic features, probes were selected based on the following criteria. Genes were classified as expressed in MEF if they had been detected on an Illumina microarray (unpublished data) with a p value of detection <0.01. Genes classified as non-expressed in MEF cells were defined if they had a p value of detection >0.5 and a signal intensity less than 0. Only those genes that contained significantly enriched Psip1/p52 and H3K36 me3 signal were used for analysis. Exonic probes were defined as those that fall within an exon - probes falling within the 5′UTR and <200 bp from TSS were excluded. Intronic regions were defined as those that fall within an intron and >200 bp from the intron start or end site. Intergenic regions probes were selected from probes that are more than 1 Kbp from either the transcriptional start sites or transcriptional end sites of a gene. The significance of differences between genomic regions was calculated using a Wilcoxon rank sum test, with a p value cutoff <0.05.

For data in Figure 6, WGA2 amplified ChIP DNA and input DNA were labeled and hybridized to a 3×720,000 probe custom microarray containing specific tiled regions encompassing 8.2 megabases of the mouse genome (Nimblegen). Array platform number is GPL14175 and the GEO accession numbers for ChIP data are; Psip1: GSM782590, H3K36me3: GSM782591, Srsf1 (Wt MEFs): GSM782592, GSM782593, Srsf1 (Psip1^−/−^ MEFs): GSM782594, GSM782595.

The median signal of replicate probes was taken prior to normalization. Data was normalized as above. Because levels of Srsf1 binding were generally quite low we used quantized correlation coefficients (QCC), which are less effected by the amount of binding signal present in the data, to determine the correlation between replicate experiments [75]. Across the entire array the QCC between Srsf1 replicates was 0.37 in wild-type cells and 0.18 in Psip1^−/−^ cells likely reflecting a loss of overall Srsf1 binding captured in the mutant cells. However, considering only the regions on the array around exons, where most Srsf1 binding is likely to be located, the QCC in wild-type cells rises to 0.5 and to 0.23 in mutant cells. Enriched probes were identified as those above a threshold defined using the upperBoundNull method from Ringo Bioconductor Package [76]. Probes above the threshold must also be located within 300 bp of 2 or more probes to be called enriched. A hypergeometric test was applied to determine significant overlap between enriched probe groups.

### Nuclear extract preparation and immunoprecipitation from NIH 3T3 cells

Nuclear extract was prepared from NIH 3T3 cells according to [77] with the following modifications: after precipitation with 1/10th vol of 4 M (NH_4_)_2_SO_4_ and mixing for 20 min, the lysate was cleared by centrifugation at 116000*g* in a TL-100 ultracentrifuge (Beckman, Mountain View, CA). The supernatant was dialyzed against 3 changes of buffer C (25 mM Hepes pH 7.6, 150 mM KCl, 12.5 mM MgCl_2_, 0.1 mM EDTA, 10% (v/v) glycerol, 1 mM DTT, 0.2 mM PMSF and complete protease inhibitors (Roche)) and flash frozen in liquid nitrogen. The extracts were quantified by Bradford assay (Bio-Rad). A total of 200 µg nuclear extract were immunoprecipitated by incubation for 45 minutes at 4°C with 5 µg rabbit IgG (Santa Cruz, sc-2027) or αPSIP1 p52/p75 (A300-847) or a-p75 (A300-348) together with 10 µl Protein A Dynal beads. After washing three times with buffer C, but containing 200 mM KCl, for 10 min each, the bound proteins were boiled in SDS sample buffer, separated on a 4–20% tris glycine polyacrylamide gel and either stained with colloidal coomassie (Invitrogen) to identify the proteins, or transferred to nitrocellulose membrane for western blotting. Individual protein bands or 1 cm^2^ gel pieces were cut and subjected to mass spectrometry analysis.

### MS/MS analysis

Excised gel pieces were treated with trypsin at 37°C and the peptides extracted with 10% formic acid. Peptides were separated using an UltiMate nanoLC (LC Packings, Amsterdam) equipped with a PepMap C18 trap & column. The eluent was sprayed into a Q-Star XL tandem mass spectrometer (Applied Biosystems, Foster City, CA) and analyzed in Information Dependent Acquisition (IDA) mode, performing 1 s of MS followed by 3 s MSMS analyses of the 2 most intense peaks seen by MS. The MS/MS data file generated was analyzed using the Mascot 2.1 search engine (Matrix Science, London, UK) against UniProt April 2009 (7966092 sequences) or NCBInr March 2010 (10530540 sequences) databases with no species restriction. The data was searched with tolerances of 0.2 Da for the precursor and fragment ions. The Mascot search results were accepted if a protein hit included at least 2 peptides with a score above the homology threshold.

### In vitro pulldowns

For p52 pulldown, T7 tagged SRSF1, SRSF3 and RG and RD mutants of SRSF1 and GFP- SRSF2, were overexpressed in 293T cells 49,78, and the cell lysates incubated with Glutathione beads coupled with p52 in GST lysis buffer. Unbound proteins were washed 5 times with the same buffer. Bound proteins were separated on 12% SDS PAGE. After transferring to nitrocellulose membrane, the proteins were probed with αT7 monoclonal antibody (Novagen) and imaged.

For histone pulldowns, 1 µg of T7 tagged SRSF1, purified from 293T cells, was incubated with T7 beads in GST lysis buffer for 1 hr at 4°C. After washing unbound proteins in same buffer, 1 µg of GST-p52 and 1 µg HeLa core histones (Active motif, cat. 53501) were added and incubated for 3 hrs. Unbound proteins were washed off 5 times with the same buffer and bound proteins were separated on 17% SDS-PAGE. After transferring to nitrocellulose membrane, the proteins were probed with αH3K36me3 antibodies and imaged. The membrane was then stripped and reprobed with αH3K9me2 and αH3K4me3 antibodies.

### Immunofluorescence

Cells grown on slides were fixed in 3% paraformaldheyde (pFa) as previously described [79] and incubated with primary antibodies; rabbit A300-847(1∶200 dilution, Bethyl laboratories,) which recognizes an epitope (a.a. 225–275) present in both p52 and p75, A300-848(1∶200, Bethyl laboratories) which recognizes only p75 (a.a. 480–530), mouse monoclonal αSc35 (1∶50, Sigma S4045). Secondary antibodies, and image capture by wide-field epifluorescence microscopy were as previously described [79]. Confocal analysis was performed using a Zeiss LSM510 confocal microscope.

### Alternative splicing microarray

Microarray analysis of alternative splicing was performed as described [53]. Five hundred ηg total RNA, isolated from primary MEFs derived from three littermates of E13.5 wild-type or *Psip1^gt/gt^* embryos [22], were used to generate sense-strand cDNA (Ambion WT expression kit #411974). Purified cDNA was fragmented and labelled with biotin-conjugated nucleotides using terminal transferase (Affymetrix, #900670). Arrays were hybridized with labelled cDNA for 16 h at 50°C in 7% dimethylsulfoxide. Washing and detection were performed in an Affymetrix Fluidics Station using standard protocols for eukaryotic targets [53]. Scanned microarrays were analyzed using ASPIRE3 (Analysis of SPlicing Isoform Reciprocity, version 3) [52], which predicts splicing changes from reciprocal sets of microarray probes that recognize either inclusion or skipping of an alternative exon. Data were quantified as the change in the fraction of exon inclusion (ΔI), where a value of 1.0 indicates a 100% increase, and −1.0 a 100% decrease in exon inclusion.

### RT–PCR

Primers corresponding to exons flanking the alternate spliced exons were designed (Table S2). 5 µg of RNA was reverse transcribed with superscript reverse transcriptase II (Invitrogen) using random primers, and each of the forward primers were labeled with ^32^P γ-ATP. PCR was performed for 24–30 cycles, and the products were separated on a 5% denaturing polyacrylamide gel and analyzed by autoradiography for 3–16 h. or separated on 1.5% agarose gel,

## Supporting Information

Figure S1 A300-847 antibody immunoprecipitates from wild-type and mutant cells. A) Silver-stained gel of immunoprecipitates with IgG, and A300-847 antibodies (anti Psip1-p52/p75) from nuclear extracts prepared from wild-type and *Psip1^gt/gt^* MEFs, 5% of the nuclear extract was loaded as input. Duplicate gel was stained with colloidal coomassie (Invitrogen), and 1 cm^2^ of the lanes corresponding to molecular weight of 25-40 KDa (indicated by boxed area) were subjected to mass spectrometry. Srsf1, Srsf5 and hnRNPm were identified from I.P with wild-type nuclear extract, but not from I.P with Psip1^gt/gt^ nuclear extract. B) Western blot of A300-847 IPs from wild-type and Psip1^gt/gt^ (A) with anti Srsf1.
(PDF)

Table S1 Peptide array quantification values of all 59 histone modifications. Cells highlighted with yellow are with specificity factor >2, and were used to generate Figure 1C.
(XLSX)

Table S2 Alternative splicing array results in Excel spread sheets (Sheet 1 includes data from all the exons on the array, Sheet 2 includes only exons which shows significant changes in alternative splicing between wild-type and *Psip1gt/gt* cells, Sheet 3 includes the annotation for the data. Related to Figure 5.
(XLS)

Table S3 Sequence of PCR primers used for RT-PCR validation of alternative splicing events in wild-type, Psip1^gt/gt^, and Psip^−/−^ cells.
(DOCX)
