# Supplementary figures and images for: Psip1/Ledgf p52 Binds Methylated Histone H3K36 and Splicing Factors and Contributes to the Regulation of Alternative Splicing
Source: PLoS Genet. 2012 May 17;8(5):e1002717. doi: 10.1371/journal.pgen.1002717 (PMC3355077; doi:10.1371/journal.pgen.1002717)

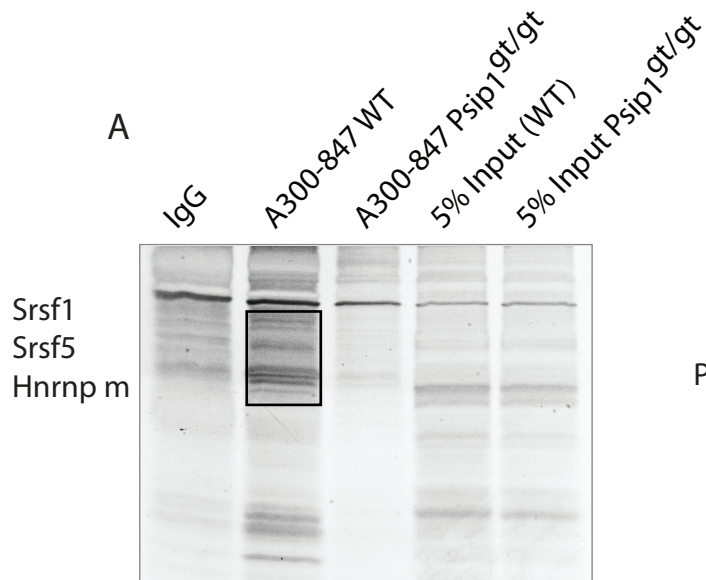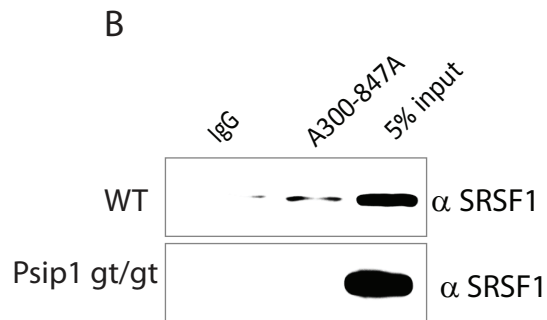

Figure S1

Supplement: Figure S1 — A300-847 antibody immunoprecipitates from wild-type and mutant cells. A) Silver-stained gel of immunoprecipitates with IgG, and A300-847 antibodies (anti Psip1-p52/p75) from nuclear extracts prepared from wild-type and Psip1gt/gt MEFs, 5% of the nuclear extract was loaded as input. Duplicate gel was stained with colloidal coomassie (Invitrogen), and 1 cm2 of the lanes corresponding to molecular weight of 25-40 KDa (indicated by boxed area) were subjected to mass spectrometry. Srsf1, Srsf5 and hnRNPm were identified from I.P with wild-type nuclear extract, but not from I.P with Psip1gt/gt nuclear extract. B) Western blot of A300-847 IPs from wild-type and Psip1gt/gt (A) with anti Srsf1. (PDF) [file pgen.1002717.s001.pdf]
