# Supplementary material for: Psip1/Ledgf p52 Binds Methylated Histone H3K36 and Splicing Factors and Contributes to the Regulation of Alternative Splicing
Source: PLoS Genet. 2012 May 17;8(5):e1002717. doi: 10.1371/journal.pgen.1002717 (PMC3355077; doi:10.1371/journal.pgen.1002717)
Supplement: Table S3 — Sequence of PCR primers used for RT-PCR validation of alternative splicing events in wild-type, Psip1gt/gt, and Psip−/− cells. (DOCX) [file pgen.1002717.s004.docx]

**Supplementary Table 3**

Sequence of PCR primers used for RT-PCR validation of alternative splicing events in Wild type and Psip1^gt/gt^cells. Psip^-/-^ MEF cells

| **Primer name** | **Sequence(5' to 3')** | **Spanning Exon** |
| --- | --- | --- |
| Ppfibp1 FP | AGAGGCTGCAGGAGAGACTG | 9 |
| Ppfibp1 RP | TGCCATCAGAGACTCCACTG | 11 |
| PTPRC FP E3 | CACTGGGTGTAGGTGTTTGC | 3 |
| PTPRC RP E7 | AAGCACTGACCCTCCAAGC | 7 |
| Rapgef6 FP | TGACTTCTGCCAACATGGAC | 21 |
| Rapgef6 RP | CAGTTTCTTGGCATTCAGCA | 23 |
| Rasgrp3 FP | ACCCTCCTTGAGCAGCAAC | 15 |
| Rasgrp3 RP | GTGACCCCAGGAAACTCAAA | 16 |
| Ogfrl1 FP | AATGACTTGAGCAACCTTCGC | 4 |
| Ogfrl1 RP | CTCGATTTCATATGTGGTTAATTC | 6 |
| Vcan con. E3 FP | GTCAAACTCCGGGCTAGTGA | 3 |
| Vcan con. E4 RP | AGCGGCAAAGTTCAGAGTGT | 4 |
| Vcan alt. E7 RP | CGGTGGCTAATGGAATGACT | Alt exon 7 |
| Vcan E2 con. FP | GCGTCTACCGATGTGATGTC | 2 |
| Vcan E3 con. RP | AGCGGCAAAGTTCAGAGTGT | 3 |
| Tpp2 con. e6FP | TATGACGATGGGAACCTGCT | 6 |
| Tpp2 con. e7RP | AGCAACTCCATTCCGTTCAG | 7 |
| Tpp2 alt. e7 alt RP | AGCATTGGTTTGCATCTGAA | Alt exon 7 |
| Tpp2 con. E1FP | CTGGACACAGGGGTCGAT | 1 |
| Tpp2 con. E2 RP | CCAATAATTTCACCATCTTTTGG | 2 |
| Diap2 con. FP | GAGATTCCCCCAGCTCTGAT | 3 |
| Diap2 con. RP | TCTTTCGCAAAGGAGCTTTC | 4 |
| Diap2 alt. RP | CAGAACTTTACTTCCAACTAT | Alt exon 5 |
| Diap2 con E8 FP | ATTTGCATTGTTGGGGAAGA | 8 |
| Diap2 con E9 RP | TCCACAATTGGTGAAAATCG | 9 |
| Sorb2 E5 FP | CCAGCATTATCATGCAGCAC | 5 |
| Sorb2 E6 RP | GGATCTATAGAGCGGGATGGA | 6 |
| Tpp2 ex 23 con FP | ATACCTAAGGGGGCAGGAC | 23 |
| Tpp2 ex 24 con RP | TTGTCGTTTTGCTGCCAGACT | 24 |
| Csnk1d FP | GGAACGAGAACGGAAAGTGA | 8 |
| Csnk1d RP | GGGGGCGTGTCACTAGTAAAG | 10 |
| Alg9 FP | GTGTATGCCATCCGCTCATA | 3 |
| Alg9 RP | GCATCATTCGACTCACATGC | 5 |
| Gapdh FP | TGGTGAAGGTCGGTGTGAACG | 1 |
| Gapdh RP | TGAGTGGAGTCATACTGG | 2 |
